# Investigating Autism-Related Symptoms in Children with Prader-Willi Syndrome: A Case Study

**DOI:** 10.3390/ijms18030517

**Published:** 2017-02-28

**Authors:** Jeffrey A. Bennett, Sandra Hodgetts, Michelle L. Mackenzie, Andrea M. Haqq, Lonnie Zwaigenbaum

**Affiliations:** 1 Department of Pediatrics, Faculty of Medicine and Dentistry, University of Alberta, 11405 87 Avenue, Edmonton, AB T6G1C9, Canada; jabennet@ualberta.ca (J.A.B.); michelle.mackenzie@ualberta.ca (M.L.M.); lonnie.zwaigenbaum@albertahealthservices.ca (L.Z.); 2 Autism Research Centre-E209, Glenrose Rehabilitation Hospital, 10230 111 Avenue, Edmonton, AB T5G 0B7, Canada; 3 Faculty of Rehabilitation Medicine, University of Alberta, 8205 114 Street, Edmonton, AB T6G 2G4, Canada; sandra.hodgetts@ualberta.ca

**Keywords:** Prader-Willi syndrome, PWS, social communication, Autism Diagnostic Observation Schedule, ADOS, autism spectrum disorder, ASD

## Abstract

Prader-Willi syndrome (PWS), a rare genetic disorder caused by the lack of expression of paternal genes from chromosome 15q11-13, has been investigated for autism spectrum disorder (ASD) symptomatology in various studies. However, previous findings have been variable, and no studies investigating ASD symptomatology in PWS have exclusively studied children. We aimed to characterize social communication functioning and other ASD-related symptoms in children with PWS, and assessed agreement across measures and rates of ASD diagnosis. Measures included the Autism Diagnostic Observation Schedule-2 (ADOS-2), the Social Communication Questionnaire (SCQ), Social Responsiveness Scale-2 (SRS-2), Social Skills Improvement System-Rating Scales (SSIS-RS), and the Vineland Adaptive Behavioral Scales-II (VABS-II). General adaptive and intellectual skills were also assessed. Clinical best estimate (CBE) diagnosis was determined by an experienced developmental pediatrician, based on history and review of all available study measures, and taking into account overall developmental level. Participants included 10 children with PWS, aged 3 to 12 years. Three of the 10 children were male and genetic subtypes were two deletion (DEL) and eight uniparental disomy (UPD) (with a total of 6 female UPD cases). Although 8 of the 10 children exceeded cut-offs on at least one of the ASD assessments, agreement between parent questionnaires (SCQ, SRS-2, SSIS-RS) and observational assessment (ADOS-2) was very poor. None of the children were assigned a CBE diagnosis of ASD, with the caveat that the risk may have been lower because of the predominance of girls in the sample. The lack of agreement between the assessments emphasizes the complexity of interpreting ASD symptom measures in children with PWS.

## 1. Introduction

Prader-Willi syndrome (PWS) is a genetic disorder caused by the absence of expression of the paternal contribution of chromosome 15q11-13. The majority of cases are due to deletion (DEL; 65%–75%) or uniparental disomy (UPD; 20%–30%), with a small minority (1%–3%) due to rare imprinting center defects [1]. The PWS phenotype includes hypotonia and failure to thrive during infancy, followed in early childhood by hyperphagia and an insatiable appetite which can lead to morbid obesity if left unchecked [1]. Cognitive disability and problem behaviors are also common and, for some families, represent greater challenges than the food-seeking behaviors [2]. 

Although variable, the PWS phenotype overlaps to some degree with autism spectrum disorder (ASD), a neurodevelopmental disorder characterized by the presence of symptoms in two core domains: social communication impairment and restricted and repetitive behaviors and interests. Indeed, the prevalence of ASD in PWS has been estimated at 26.7% based on exceeding clinical cut-points on relevant ASD assessments [3]. Also, ASD in the UPD genetic subtype (35.3%; two maternal copies of chromosome 15 present) is almost twice as common as in the DEL subtype (18.3%) [3]. This may be partially due to a genetic finding that overexpression of chromosome 15 is associated with higher rates of ASD [4]. The estimated prevalence of ASD in PWS is significantly higher than the current prevalence estimate of about 1.5% for ASD in the general population [5]. To receive an ASD diagnosis, symptoms in both domains must be present during the early developmental period; however, they may not be fully recognized until a child is older and social demands exceed their capacity [1]. Nevertheless, early social communication impairments (including reduced gazing towards faces and directed vocalizations [6], and reduced response to name being called [7]) in children who later receive a diagnosis of ASD have been reported as early as 12 months. Indeed, ASD diagnosis can be reliably made by 18 to 24 months in a clinical setting. Earlier recognition of ASD is important as studies have demonstrated that early intervention for ASD yields more favorable outcomes [8]. 

Despite a recent increase of research investigating symptoms of ASD in PWS, no studies have focused on young children with PWS. Eight years of age is the youngest mean age in a study investigating ASD in individuals with PWS [9]. The expression of ASD in PWS may change over development, with two studies reporting more prominent symptoms in adolescents and adults with PWS compared to younger children. Lo et al. [10] reported that none of the 22 children with PWS, ages 7–9, exceeded the cut-off for ASD on the Diagnostic Interview for Social and Communication Disorders (DISCO); however, 24 of 44 of the individuals aged 10–17 years old exceeded the cut-off for ASD. Additionally, Akefeldt and Gillberg [11] reported lower average scores (3.4; SD = 4.4) in toddlers with PWS (mean age = 2.1 years; range = 0.8–3.7 years) compared to the older individuals (19.1; SD = 10.7) with PWS (mean age 18.4 years; range = 4.2–36.3 years) on the Autism Spectrum Screening Questionnaire (ASSQ) [12]. However, this tool was designed to assess children ages 6 to 17 years old with normal intelligence to mild cognitive disability, raising concerns about the validity of the ASSQ in this sample. 

Assessments commonly used to investigate ASD symptomatology and adaptive functioning in individuals with PWS include the Social Responsiveness Scale [13,14], Social Communication Questionnaire [15,16], and Vineland Adaptive Behavior Scales [13,17]. A recent systematic review [3] found that few studies investigating ASD in PWS have included the Autism Diagnostic Observation Schedule (ADOS) [18] or the Autism Diagnostic Interview-Revised (ADI-R) [19], considered to be gold standard measures of ASD symptoms based on excellent sensitivity and specificity in differentiating ASD from other developmental disorders [20]. Moreover, rates of ASD reported in PWS are generally not based on ICD or DSM-based clinical diagnoses, but are rather based on clinical cut-offs on proxy measures such as the Social Communication Questionnaire (SCQ) [3]. This is problematic, as no single measure can be used as a proxy for diagnosis. 

The primary goal of this study was to characterize ASD symptoms in children ages 3 to 12 with PWS using the ADOS, 2nd edition (ADOS-2) [21], and other standardized ASD assessment tools. As a secondary objective, we investigated other aspects of social-communication development in children with PWS, and compared the agreement between various ASD assessment tools and categorical ASD diagnosis by clinical best estimate (CBE). Based on previous studies, we hypothesized elevated rates of ASD symptomatology in children with PWS, as has been found in adolescents and adults with PWS. We also expected ASD symptoms to be present in some children with PWS who do not have a diagnosis of ASD. Furthermore, we expected a moderate agreement between the various ASD tools used to assess ASD symptomatology in children with PWS. 

## 2. Results

### 2.1. Demographics 

There were a total of 10 participants, all of whom resided in western Canada (Alberta (*n* = 8); British Columbia (*n* = 1); Saskatchewan (*n* = 1)). Nine of the ten patients with PWS in the age range who were contacted directly through a regional specialty clinic agreed to participate in the study. One additional child was recruited through a provincial PWS group. The cohort included seven females and three males, with a mean age of 6.77 years old (SD = 2.85). Participant genetic subtype was confirmed by genetic testing completed previously, or through confirmation of genetic testing by the participants’ health-care provider. Only two participants were the DEL subtype; the other eight were the UPD subtype. Each participant completed all of the assessments. Table 1 provides a summary of the study results, including the mean, standard error of the mean, and standard deviation, as well as the minimum and maximum scores attained for all measures. The average full scale IQ (FSIQ) was 63.38 (SD = 17.42), which falls within the range for individuals with PWS reported in a review by Cassidy and colleagues [1]. A negative correlation between age and FSIQ was found (Spearman’s *ρ* = −0.815, *p* = 0.004). 

### 2.2. Assessments

Table 2 gives detailed results for every participant on each of the ASD assessments, including an indication of which scores exceeded clinical cut-offs. In total, three of 10 children (all 3 female UPD) scored above the cut-off for ASD on the ADOS-2 symptom severity score. However, two of those three children (P8 and P9) scored 0 in the restricted and repetitive behaviors (RRB) domain, which would suggest that ASD is not present, as under DSM-5 observable symptoms are expected in both the social affect (SA) and RRB domains. 

#### 2.2.1. ASD Cut-Off Scores

Five children exceeded the cut-off SRS-2 score associated with ASD in the general population, four of whom also exceeded cut-off on the SCQ. With respect to the separate domains, social motivation showed the least impairment (T-score = 56.10), while the RRB domain showed the most impairment (T-score = 65.80). No differences were detected among SRS-2 domain means, based on a non-parametric Kruskal-Wallis Test. It should be noted that, although a modified cut-off score of 12 was used for the SCQ, using the original cut-off score of 15 did not add meaningful clarification to the overall scores or patterns of agreement between assessments. Six of 10 children (1 male DEL, 1 male DEL, 4 female UPD) had above average ASD symptoms on the SSIS-RS ASD subscale. These six children also exceeded cut-off on the SCQ and/or the SRS-2. 

#### 2.2.2. Clinical Best Estimate

None of the participants met clinical best estimate (CBE) criteria for ASD based on DSM-5, largely because of inconsistency across measures. Detailed case descriptions of each participant (P) are provided below. To maintain confidentiality, age and gender cannot be reported, although the participants are ordered from youngest (P1) to oldest (P10) to provide a sense of relative age. Each profile describes the unique or extreme findings from each child.

#### 2.2.3. Case Study Descriptions

P1 (female DEL) was the youngest individual in our study. The assessment results from this participant were not indicative of ASD. The Restricted or Repetitive Behaviors and Interests (RRB) sections on both the ADOS-2 and SRS-2 confirmed mild impairment in this symptom domain, but the ADOS-SA domain was low at a severity score of 2. The SSIS-RS percentiles and Vineland-II scores were average, with the exception of the Vineland-II motor skills domain, which was more than one standard deviation below the normative standard. 

P2 (male UPD) did not exceed cut-off for ASD on any of the assessments. Again, the SSIS-RS and Vineland-II results were all close to the normative standard, with the exception of motor skills on the Vineland-II. 

P3 (female UPD) had parent-reported symptoms from the three ASD questionnaires (SCQ, SRS-2, and SSIS-RS) that were the lowest in the entire sample. Indeed, she had among the most favourable scores on the SSIS-RS and Vineland-II of all participants, scoring above the normative standard on all domains except Vineland-II socialization and Vineland-II motor skills domains, both of which were less than one standard deviation below age-related norms. In contrast, P3 is the only child who exceeded the ASD threshold on both the SA and RRB domains of the ADOS-2, albeit with an overall severity score right at the cut-off associated with ASD. 

P4 (male UPD) had SCQ and SSIS-RS ASD subdomain scores that exceeded the cut-off for ASD, while his SRS-2 score was one point below cut-off. The SRS-2 domains that elevated the overall scores included social awareness, social cognition, and RRBs. He also scored below the sample mean on the social skills subdomain of the SSIS-RS (18th percentile), and higher on the problem behaviors subdomain (90th percentile). However, he was assessed as having social-communicative symptoms well below the level associated with ASD (ADOS-SA severity score = 2, and overall severity score = 2), although RRB symptoms were observed. As well, Vineland-II social and communicative domains were rated as close to average. 

P5 (female UPD) had similar results as P4: low ADOS-2 scores, despite exceeding cut-off for ASD on two of the three parent-report questionnaires. Critically, the SCQ score was three points below the cut-off associated with ASD and the SRS-2 overall score was only two points above cut-off. She also scored low on the social skills domain of the SSIS-RS (14th percentile), and very high on the problem behaviors domain (98th percentile). Additionally, the Vineland-II scores were all below the sample mean, with motor skills being the most affected. 

P6 (female UPD) was assessed as having social-communication symptoms below the level associated with ASD (severity score = 3) on the ADOS, despite RRB symptoms (severity score = 6). However, she was rated as having high levels of symptoms on all three questionnaires. The SRS-2 scores were especially high, with the overall score one point below the severe range. These scores were largely driven by the RRB and the social cognition subdomains, both of which fell in the severe range; comparatively, the social motivation subdomain only showed mild impairment. Additionally, the SSIS-RS social skills (8th percentile) and problem behavior (97th percentile) were both indicative of significant social impairments. Despite the high scores on the questionnaires, P6 scored within one SD of age norms on the communication and daily living skills domains on the Vineland-II, although more than one standard deviation lower on the socialization and motor skills domains. Full scale IQ was 56, so clinically, social impairments were felt to relate, at least in part, to intellectual impairments.

P7 (male DEL) also scored below the clinical threshold on the ADOS-2, with an overall severity score of 3, despite symptoms observed in both domains. He exceeded the cut-off for ASD on all three questionnaires. On the SSIS-RS, this child was rated at the 2nd percentile on the social skills domain, >99th percentile for the problem behaviors domain, and the highest raw score on the ASD subdomain. The participant’s SCQ score was also elevated. Interestingly, all of the subdomains on the SRS-2 were extremely similar (scores ranged from 65–67), with the exception of social cognition, which was below cut-off. 

P8 (female UPD) had an overall symptom severity rating of 5, above the level associated with ASD, although with no symptoms observed in the RRB domain (severity score = 0). The parent report on the SRS-2 did suggest severe impairments in the RRB subdomain, with overall SRS-2 score at the upper end of the moderate range. SSIS-RS social skills (4th percentile) and problem behaviors (96th percentile) were also indicative of social impairment, and the ASD subscale score was very high as well. She scored over two standard deviations below the normative standard on the communication domain of the Vineland-II, and had slightly higher scores in the other three domains. Other factors taken in consideration in the CBE process included cognitive delay (FSIQ = 47). 

P9 (female UPD) had high levels of symptoms rated on the SA domain of the ADOS (severity score = 7), but no observed RRB symptoms (severity score = 0). As well, she had among the lowest scores on the SSIS-RS ASD domain, the SCQ, and the SRS-2, including on the RRB domain. The only SRS-2 subdomain that exceeded cut-off for ASD was social motivation, albeit by one point. 

P10 (female UPD) had symptoms rated in both SA (severity score = 3) and RRB. (severity score = 7) of the ADOS, although the overall severity was subthreshold for ASD (severity score = 3). Scores were also elevated on the SCQ and SRS-2. This child’s SSIS-RS ASD score was also well above cut-off, and the SSIS-RS social skills (2nd percentile) and problem behaviors (98th percentile) also showed significant impairment. She also had the lowest adaptive behavior composite score on the Vineland-II, with the socialization and communication scores almost three standard deviations below standard. However, the clinician responsible for CBE diagnosis concluded that impairments in socialization and communication were largely attributable to intellectual disability (FSIQ = 40), and that this child did not meet DSM-5 criteria for ASD.

### 2.3. Agreement among Measures

The three questionnaires (SRS-2, SCQ, and SSIS-RS) all had acceptable agreement with one another (see Table 3). Cohen’s κ for the SCQ and SSIS-RS, as well as for the SRS-2 and SSIS-RS, was calculated to be 0.80 (SE = 0.19), and κ for the SCQ and SRS-2 was calculated to be 0.60 (SE = 0.25). Notably, a total of six children exceeded the cut-off on the SSIS-RS; 4 of 6 children exceeded the cut-off on both the SRS-2 and SCQ, a fifth on the SRS-2 but not the SCQ, and the sixth on the SCQ but not the SRS-2. However, agreement between the three questionnaires and the ADOS-2 was poor. Cohen’s κ for the ADOS-2 vs. the SCQ, SRS-2, and SSRI-RS were calculated to be −0.20 (SE = 0.28), −0.20 (SE = 0.28), and −0.30 (SE = 0.28), respectively.

## 3. Discussion

Our main findings were that (1) ASD symptomatology and social competence in children with PWS were highly variable; (2) While there was moderate to high agreement among parent-report measures of ASD symptoms and social behaviors (κ = 0.6–0.8), agreement between these measures and ASD symptom severity as observed on the ADOS-2 was poor (κ < 0.0) (see Table 3). Six of the 10 children (1 male DEL, 1 male DEL, 4 female UPD) exceeded the cut-off for ASD on at least one of the parent-report measures (and 4 of 10 on all three). In contrast, 3 of 10 (all female UPD) had observable symptom severity at the level associated with ASD diagnosis on the ADOS-2, only one of whom had elevated symptoms in both domains. Moreover, only 1 of the 3 children with elevated ASD symptom severity on the ADOS-2 met the scoring cut-off suggestive of ASD on *any* of the parent report measures (not the same child with elevated symptoms on both ADOS-2 domains). Thus, while most (8 of 10) participants had evidence of ASD symptoms on at least one study measure, there was poor agreement between measures and thus, inconsistent findings that complicated clinical diagnostic judgement. Indeed, none of the participants were assigned a clinical diagnosis of ASD. One child (P8) with elevated symptoms on both the ADOS-2 (albeit not both domains) and the parent report measures had an FSIQ of 47, and thus, the clinical presentation was complicated by intellectual disability. This child’s inability to perform numerous tasks made the interpretation of social skills deficits more challenging. Although the clinical diagnostic status of this child may be equivocal, the pattern of scoring overall on study measures indicated considerable variability in profile, poor agreement between parent-report measures, and direct behavioral observation (i.e., using the ADOS-2), and raises important questions about the clinical interpretation and even the validity of available ASD symptom measures in this population. Finally, there was a negative correlation between age and FSIQ (Spearman’s *ρ* = −0.815, *p* = 0.004 in this small sample). 

In our study, sample means on ASD assessments in children with PWS were less indicative of ASD than have been previously reported in adolescents and adults with PWS. For example, Zyga et al. [14] and Dimitropoulos et al. [13] used the SRS to assess samples with a mean age over 10 years of age, and found average scores of 82.18 and 76.31, respectively. Both of these scores fall in the severe range on the SRS-2, whereas the average score from our population was 62.70, which falls in the mild range. Dimitropoulos et al. [13] and Milner et al. [17] also used the Vineland-II in their samples of adolescents and adults with PWS, and reported average composite scores of 65.15 and 62.60, respectively. Meanwhile, our sample had an average score of 80.40, over one standard deviation above results from these previously published studies. Lastly, Zyga et al. [14] reported that 8 of 14 adolescents (57.14%) met criteria for ASD on the ADOS, whereas only 3 of 10 (30%) from our cohort met criteria (actually only 1 of 10 (10%), considering that P8 and P9 did not pass cut-off on the RRB domain). These findings indicate interesting differences in ASD symptoms and adaptive functioning between age groups (children vs. adolescents and adults). However, other potential confounders, such as growth hormone treatment or exposure to other interventions, cannot be ruled out.

There were marked discrepancies between direct observational assessment and parent report in the assessment of ASD in PWS in our study. Notably, previous research in non-PWS samples indicates that both parent reports and observational data are essential to valid diagnostic assessment for ASD [22]. In a recent systematic review of ASD in PWS, Bennett et al. [3] reported that most studies investigating ASD in PWS are based on single measures, with parent reports more common than observational assessment (i.e., ADOS-2). Given that parent reports may be less resource-intensive and require less specialized training, they may be more feasible to administer in a clinical program serving a rare population such as PWS. However, given the limited agreement across parent reports and direct observational measures in our sample, further research may be needed to verify which assessments are most accurate and informative in the clinical diagnostic assessment of ASD in PWS. 

Another possible explanation for the discrepancy between the results from the ADOS-2 and the parent-report assessments could be the distinction between what a child ‘can do’ and ‘typically does’. The AODS-2 is a 30 to 45-minute observational assessment designed to elicit certain responses from children. Since some children with PWS are more comfortable associating with adults than same-age peers, the ADOS-2 interaction potentially provides a more comfortable situation than their usual social experiences. Parent-report measures, on the other hand, focus on the frequency of the child’s social behaviors in their real-life settings, which may be influenced by both ability and opportunities/contexts. Another possible explanation for poor agreement between the SCQ questionnaire and ADOS-2 is that the restrictive repetitive behaviors on the SCQ (which are more prevalent in PWS) are not as heavily weighted on the ADOS-2. However, it would be difficult to know whether any of these possibilities are valid without further research to probe discrepancies between the various ASD assessments employed in this study.

Lack of agreement between ASD assessment measures has been reported in studies investigating ASD in other genetic syndromes, specifically Fragile-X syndrome [23]. These studies indicate poor agreement between the ADOS-2 and SCQ (Cohen’s κ = 0.33 for girls, 0.13 for boys). Additionally, using a combination of the ADI-R, ADOS, and DSM-IV criteria, Harris et al. [24] reported that in a group of 63 males with Fragile X syndrome, 15 participants (24%) met criteria for ASD on all three assessments, while an additional 28 individuals (44%) met criteria on only one or two of the assessments. One potential explanation for these findings is that each genetic syndrome manifests its own set of complex behaviors which are not typical in the general population, some of which may overlap with ASD, even when the full ASD phenotype is not present [25,26]. The overlap in phenotype places individuals with a genetic syndrome closer to ASD cut-off scores on particular measures than typically developing individuals, even in the absence of an ASD diagnosis. Another contributor to higher scores on ASD assessments may be the degree of intellectual disability. Indeed, the SCQ manual [27] mentions that non-ASD individuals with lower IQ (50–69) obtained higher scores on the SCQ (11.40; SD = 5.87), which is quite similar to the results in our study (mean IQ = 63.38; mean SCQ score = 11.90). The SRS-2 manual [28] gives similar caution to its use in individuals with an IQ less than 70. Both overlapping phenotype and intellectual disability may affect the ability for these assessments to reliably detect ASD in PWS. 

Limitations to this study include the sample demographics, limited sample size, and cross-sectional design. The limited number of children available for the study resulted in uneven distribution in both gender and genetic subtype. Our study included three boys and seven girls, whereas the gender distribution in the PWS population is roughly equal. Given that ASD is recognized four times more in males than females [5], the high number of females in the study may have reduced the likelihood of ASD diagnosis. The ratio of 7:3 in favor of girls (in our study) is not an extreme variation, which would be expected about 17% of the time based on the binomial distribution. As well, the presence of ASD symptoms on the questionnaire measures and the ADOS did not seem to predominantly characterize males, albeit with only 3 in the sample. ASD in general is less common in females, although sex differences in ASD symptoms are generally not reported in PWS studies [3]. As well, although recruitment involved every known child with PWS in our study age-range, there was an unexpected distribution of eight UPD and two DEL cases in our sample. It has been reported that the UPD genetic subtype is associated with a higher level of ASD symptoms than the DEL subtype [3,29,30]. This sample characteristic might have been expected to lead to a higher level of ASD symptomatology. Notably, 1 of 2 (50%) DEL cases exceeded the cut-off on multiple ASD assessments, whereas 7 of 8 (88%) UPD exceeded the cut-off on at least one of the ASD assessments. It is important to note that it is unlikely that recruitment was biased to include more UPD children, given that 9 of 10 parents initially contacted agreed to participate in the study, without prior knowledge of genetic subtype from those contacting the participants. Notably, within the DEL subtype, there are two types of deletions: the longer type 1, and relatively shorter type 2. Some studies have found differences between type 1 and type 2, with type 1 typically showing greater overall impairment as well as ASD-related impairments [17,31,32,33,34]. However, we were unable to distinguish between the 2 types in our study, as the sample population had not received the necessary genotyping. Individuals with truncating mutations on the paternal allele of MAGEL2, a gene within the PWS domain, were recently found to exhibit both features of PWS and autism [35]. The overall implication is that our current limited sample was predominantly female UPD which may have influenced ASD symptom levels, although the two factors (sex and genetic subtype) would have been expected to shift the distribution in opposite directions. Our subsample of 6 girls with UPD is a relatively large grouping of this rarer subtype, and may provide a useful detailed clinical reference for other such patients. Critically, this study was originally intended to make group comparisons between UPD, DEL, and an ASD comparison group, but lacked an available adequate sample size due to lack of children with PWS in the region. Although descriptive studies are able to provide a wealth of information regarding smaller samples in order to generate hypotheses and ideas, they lack the statistical power to compare results to other groups, such as an ASD comparison group. A matched control group could have helped shed further insight into both the ASD symptoms and the relative strengths and weaknesses identified in this study. As well, our clinical best estimate (CBE) was done by an experienced developmental paediatrician, who reviewed all results, watched videotapes of the ADOS-2 assessments and met with individual patients for further discussion. However, there was no second rater that would have been necessary to determine reliability of the CBE in this study. Finally, the negative correlation between age and IQ in children with PWS is of interest, but is difficult to interpret in this small sample, which does not allow us to parse potential confounding effects of sex, genetic subtype, and obesity. A longitudinal design would also be helpful to discriminate possible factors involved in the negative correlation between age and IQ, and to determine if ASD symptoms in PWS are exacerbated with age.

## 4. Materials and Methods 

### 4.1. Study Design

This cross-sectional study was conducted in Edmonton, AB, Canada. Individuals aged 3 to 12 with PWS were recruited for this study from local and regional care providers, as well as provincial PWS groups. The ratio of UPD to deletion cases of PWS (and girls versus boys) was the result of random chance; those who met study inclusion criteria from the local area were recruited. Parents agreed to receive a report of their child’s performance and to be referred for further assessment and treatment if indicated clinically by the findings. Ethical approval was received from the local Health Research Ethics Board Health Panel (Study ID: Pro00051250; Approved 7 January 2015) at the University of Alberta. Parents gave informed consent for their children prior to participation in this study.

### 4.2. Assessments

All assessments were administered by experienced research psychometrists, who had been trained to reliability on each of the measures (including research reliability on the ADOS-2). Data collection was blinded to genetic subtype to avoid bias. A developmental paediatrician with 20 years experience in ASD diagnosis (LZ) reviewed case files and ADOS videos to determine whether criteria were met for ASD for each participant (hereafter, ‘clinical best estimate’ diagnosis). 

#### 4.2.1. Autism Diagnostic Observation Schedule-2

The Autism Diagnostic Observation Schedule, 2nd edition (ADOS-2) [21] is a semi-structured interactive assessment that provides an opportunity to observe behaviors relevant to ASD diagnosis. Items are scored from 0–3 or 0–2, with ‘0’ representing a continuum of behavior not generally associated with ASD; a code of ‘1’ generally indicates mild impairment of a nature that may be observed in persons with ASD; a code of ‘2’ indicates definite impairment in that area; a code of ‘3’ represents more profound impairment, although for the purpose of the scoring algorithm, scores of ‘3’ are converted to ‘2’. The ADOS-2 provides a total score for social affect (SA) and restricted and repetitive behaviors (RRB) symptoms, each of which can be translated into a standardized severity score on a scale of 1 to 10. There is also an overall severity score, with ≥4 indicative of ASD. Because the severity of SA symptoms is rated based on ten algorithm items and RRB severity is based on four items, the overall severity score is more heavily weighted towards the SA domain. Additionally, since the RRB domain only consists of 4 algorithm items, if all items are scored ‘0’, the severity is 0, whereas any item scored ‘1’ leads to a severity rating of 5. Thus, the RRB severity scores are highly sensitive to scoring on individual items, and must be interpreted with caution. The ADOS-2 has been validated for ages 12 months to adulthood. One of 5 modules is administered, based on age and verbal fluency; Only modules 1 to 3 were administered in our study. Module 1 is designed for individuals with no speech or single words; Module 2 is for individuals with phrase speech; and Module 3 is for individuals with fluent speech and typically less than 14 years of age. 

#### 4.2.2. Social Communication Questionnaire

The Social Communication Questionnaire (SCQ) [27] is a 40-item parent questionnaire used to screen for autistic symptomatology; it is derived from the Autism Diagnostic Interview-Revised (ADI-R), a semi-structured interview used in ASD diagnosis [19]. The SCQ, which uses a yes/no parent response form, was chosen over the ADI-R to be more time-feasible for parents (10 minutes for the SCQ vs. 2 h for the ADI-R). Raw scores of 15 or greater are indicative of ASD. A study released after the publication of the SCQ indicates that a cut-off of 12 greatly improves the sensitivity of the assessment when used in combination with the ADOS, particularly for younger children [36].

#### 4.2.3. Social Responsiveness Scale-2

The Social Responsiveness Scale, 2nd edition (SRS-2) [28] is a questionnaire completed by a primary caregiver and/or a teacher that provides an overall rating of social impairment as well as scores on 5 ASD-specific subdomains: (1) Social Awareness; (2) Social Cognition; (3) Social Communication; (4) Social Motivation; and (5) Restricted or Repetitive Behaviors (RRBs). It is designed to assess individuals from age 2.5 years to adulthood. Total scores on the SRS-2 are standardized as T-scores, based on age and gender, and are further separated into four levels: <60 (Within normal limits; generally not associated with ASD); 60 to 65 (Mild range; indicates deficiencies in reciprocal social behavior that may lead to mild to moderate interference with everyday social interactions); 66 to 75 (Moderate range; indicates deficiencies in reciprocal social interaction that lead to substantial interference with everyday social interaction, and are typical for children with ASD of moderate severity); and >75 (Severe range; indicates deficiencies in reciprocal social interaction that lead to severe interference with everyday social interaction, and are strongly associated with a clinical diagnosis of ASD).

#### 4.2.4. Social Skills Improvement System-Rating Scales

The Social Skills Improvement System-Rating Scales (SSIS-RS) [37] was selected to provide information on a broad range of social behaviours; i.e., not only those specifically impaired in ASD. It is a parent questionnaire that can be administered for children between the ages of 3 and 18. The SSIS-RS provides percentiles for social skills (higher scores indicate more advanced social skills) as well as problem behaviors (higher scores indicate increased problem behaviors), normed by gender and age. There is also a subscale to indicate ASD symptoms (rated as below average, average, or above average). Although the SSIS-RS has not previously been reported in a PWS population, it has been widely used in the ASD population and determined to be a valid research tool for measuring social skills [38]. Furthermore, this questionnaire also assesses non-ASD related social impairments that might be present in PWS.

#### 4.2.5. Vineland Adaptive Behavior Scales-II

The Vineland Adaptive Behavior Scales, 2nd edition (Vineland-II) [39] is a parent interview that quantifies their child’s current adaptive behaviors. It provides a composite score, plus sub-scores in the following domains: (1) Daily Living Skills; (2) Socialization; (3) Communication; and (4) Motor Skills. Scores are based on a standardized scale with a mean of 100, and standard deviation of 15. It has been validated for all ages and specifically for individuals with ASD [40]. 

#### 4.2.6. Wechsler Preschool and Primary Scale of Intelligence-III/ Wechsler Intelligence Scale for Children-IV

The Wechsler Preschool and Primary Scale of Intelligence, 3rd edition (WPPSI-III) [41] and Wechsler Intelligence Scale for Children, 4th edition (WISC-IV) [42] are individually administered measures of cognitive abilities that yield full-scale IQ. The WPPSI-III has been validated in children from age 2 to 7 years, and the WICS-IV has been validated in children from age 6 to 16 years. Both the WPSSI-III and WISC-IV have been used previously to assess patients with PWS and ASD [13,14,43].

### 4.3. Analytic Approach

Descriptive data from the ADOS-2, the 3 questionnaires (SCQ, SRS-2, and SSIS-RS), and the Vineland-II were provided for each child. Clinical best estimate (CBE) ASD diagnosis included a video review of each participant’s ADOS-2, review of the questionnaires, and in person meeting with the parent and child. Any test result exceeding previously recommended ASD clinical cut-offs were considered in relation to CBE. Summary statistics were calculated for each assessment, including mean, standard deviation, standard error of the mean, and range. Additional analyses were performed using SPSS version 22 to compare performance among domains within measures. Experiment-wise alpha was set at *p* < 0.05 for all statistical analyses, which were completed using non-parametric tests due to the small sample size and associated non-normal distribution of study measures across our sample.

## 5. Conclusions

Our data suggest variable ASD symptomatology in children with PWS, with caveats related to the sex and genetic subtype composition of our small sample. Further research is necessary to identify the most appropriate tools to assess ASD in PWS. In particular, the disparity in agreement between direct observational assessment and parent reports warrants investigation into appropriate assessment of ASD in children with PWS. 

## Figures and Tables

**Table 1:** Results from assessments, given as mean (SE; SD) and range.

	Mean (SE; SD)	Min–Max
Age	6.77 (2.85)	3.42–11.75
# (%) male	3 (30)	–
# (%) UPD	8 (80)	–
FSIQ	64.70 (5.05; 15.96)	40–92
ADOS-2 Severity score	3.00 (0.42; 1.33)	1–5
ADOS-2 SA Severity score	3.50 (0.56; 1.78)	2–7
ADOS-2 RRB Severity score	4.70 (0.80; 2.54)	0–7
SCQ raw score	11.90 (2.20; 6.95)	5–21
SRS-2 Overall T-score	62.70 (3.98; 12.56)	43–85
SRS-2: Social Awareness T-score	61.60 (4.16; 13.16)	43–81
SRS-2: Social Cognition T-score	61.00 (4.83; 15.27)	40–94
SRS-2: Social Communication T-score	61.70 (4.04; 12.78)	43–86
SRS-2: Social Motivation T-score	56.10 (2.47; 7.81)	43–67
SRS-2: RRB T-score	65.80 (4.20; 13.28)	48–88
SSIS-RS: Social skills percentile	23.10 (7.02; 22.18)	2–64
SSIS-RS: Problem behaviors percentile	80.00 (7.06; 22.32)	42–99
SSIS-RS: ASD raw score	19.00 (2.53; 8.01)	8–31
Vineland-II Composite standard score	80.40 (3.57; 11.29)	60–96
Vineland-II: Communication standard score	85.20 (4.60; 14.56)	59–100
Vineland-II: Daily Living Skills standard score	86.50 (3.60; 11.38)	68–101
Vineland-II: Socialization standard score	83.20 (4.79; 15.14)	57–108
Vineland-II: Motor standard score	77.20 (2.59; 8.19)	67–91

*n* = 10 for all assessments; SE = Standard Error (of the mean); SD = Standard Deviation (of the sample); FSIQ = Full-scale IQ (based on WPPSI/WISC scores); ADOS-2 = Autism Diagnostic Observation Schedule-2; SA = Social Affect; RRB = Restricted and Repetitive Behaviors; SRS-2 = Social Responsiveness Scale-2; SCQ = Social Communication Questionnaire; SSIS-RS = Social Skills Improvement System-Rating Scales.

**Table 2:** Individual participant score profiles.

ID	Sub-Type	Sex	FSIQ	ADOS Module	ADOS SA	ADOS RRB	ADOS Overall	SCQ	SRS AWR	SRS COG	SRS COM	SRS MOT	SRS RRB	SRS Overall	SSIS SS	SSIS PB	SSIS ASD	CBE
1	DEL	F	92	2	2	6	2	6	54	56	55	51	62	56	25	53	11	N
2	UPD	M	63	2	2	5	1	6	43	54	48	57	56	52	50	56	16	N
3	UPD	F	77	2	4	6	*4*	5	49	40	43	43	48	43	64	42	8	N
4	UPD	M	82	2	2	6	2	*14*	66	63	57	47	64	59	18	90	*21*	N
5	UPD	F	69	2	2	6	2	9	57	53	64	54	58	*62*	14	98	*20*	N
6	UPD	F	56	2	3	6	3	*13*	68	77	71	64	82	*75*	8	97	*26*	N
7	DEL	M	65	3	4	5	3	*20*	67	55	65	67	66	*65*	2	99	*31*	N
8	UPD	F	47	1	6	0	*5*	*14*	81	67	73	64	80	*75*	4	96	*21*	N
9	UPD	F	56	3	7	0	*5*	6	50	51	55	60	54	55	44	71	8	N
10	UPD	F	40	3	3	7	3	*26*	81	94	86	54	88	*85*	2	98	*23*	N

Subtype: DEL = deletion ; UPD = uniparental disomy; Sex: M=Male, F = Female; ADOS-SA = ADOS-2 Social Affect Severity Score; ADOS-RRB = ADOS-2 Restricted or Repetitive Behavior Severity Score; SCQ = SCQ Raw Score; SRS-AWR = SRS-2 Social Awareness T-score; SRS-COG = SRS-2 Social Cognition T-score; SRS-COM = SRS-2 Social Communication T-score; SRS-MOT = SRS-2 Social Motivation T-score; SRS-RRB = SRS-2 Restricted or Repetitive Behaviors T-score; SSIS-SS = SSIS-RS Social Skills Percentile; SSIS-PB = SSIS-RS Problem Behaviors Percentile; SSIS-ASD = SSIS-RS ASD Subscale Raw; CBE = Clinical Best Estimate; ADOS-2: scores of >3 are associated with ASD; SCQ: scores of ≥12 are associated with ASD; SRS-2: scores <60 are not associated with ASD, 60–65 are associated with mild to moderate impairment in social responsiveness, 66–75 are associated with substantial impairment in social responsiveness and ASD, >75 are associated with severe impairment in social responsiveness and ASD; SSIS-RS: overall score for the following age ranges represents the average amount of ASD behaviors; any score above or below these ranges indicate above or below average ASD symptoms, respectively: age 3–5 years: 4–16; age 5–12 years: 3–14; CBE: N = does not qualify for ASD diagnosis, Y = does qualify for ASD diagnosis; * Scores that exceed cut-off for ASD are ***bolded and italicized*** *. Note: The individuals were ordered from youngest (ID = 1) to oldest (ID = 10) in order to give a sense of participant age (range = 3–12 years); to maintain anonymity (due to the rarity of PWS), participant ages not listed; rather, ordered by age.

**Table 3:** Agreement among Measures.

Measures	Kohen’s κ	Standard Error
SCQ/SRS-2	0.60	0.25
SCQ/SSIS-RS	0.80	0.19
SRS-2/SSIS-RS	0.80	0.19
SCQ/ADOS-2	−0.20	0.28
SRS-2/ADOS-2	−0.20	0.28
SSIS-RS/ADOS-2	−0.30	0.28

## Abbreviations

ADI-R	autism diagnostic interview-revised
ADOS-2	autism diagnostic observation schedule, 2nd edition
ASD	autism spectrum disorder
CBE	clinical best estimate
DEL	deletion (genetic subtype)
PWS:	Prader-Willi syndrome
RRB	restricted or repetitive behaviors
SCQ	Social Communication Questionnaire
SRS-2	Social Responsiveness Scale-2
SSIS-RS	Social Skills Improvement System-Rating Scales
UPD	uniparental disomy (genetic subtype)
